# Adherence to Social Distancing Measures for Controlling COVID-19 Pandemic: Successful Lesson From Vietnam

**DOI:** 10.3389/fpubh.2020.589900

**Published:** 2020-11-16

**Authors:** Hoang-Long Vo, Hao Anh Si Nguyen, Khanh Ngoc Nguyen, Huong Lan Thi Nguyen, Hien Thi Nguyen, Long Hoang Nguyen, Giang Thu Vu, Huong Thi Le

**Affiliations:** ^1^Institute for Preventive Medicine and Public Health, Hanoi Medical University, Hanoi, Vietnam; ^2^Institute for Global Health Innovations, Duy Tan University, Da Nang, Vietnam; ^3^Faculty of Nursing, Duy Tan University, Da Nang, Vietnam; ^4^Institute of Health Economics and Technology, Hanoi, Vietnam; ^5^Center of Excellence in Evidence-based Medicine, Nguyen Tat Thanh University, Ho Chi Minh City, Vietnam

**Keywords:** COVID-19, SARS-CoV-2, adherence, social distancing, Vietnam

Social distancing measure has been considered an effective policy response across nations to mitigate the spread of the COVID-19 pandemic (1). Given the matter that SARS-CoV-2 transmits via close contact, social distancing requires individuals to keep a distance of at least 6 feet from people who do not belong to their households (1). Vietnam has achieved initial results in flattening the curve and slowing the spread of COVID-19 transmission in the community, which is mainly attributable to a high-level adherence of Vietnamese with social distancing measures, accompanied with contact tracing, mass testing, and mandatory isolation (2).

Social distancing measure had been implemented in Vietnam, a country having a long borderline with China, from a very early phase as one of the precautionary measures since the first case was detected on 22 January (Figure 1). On 31 January 2020, the Prime Minister issued the Directive 06/CT-TTg (3) to enforce banning, suspending, or narrowing traditional festivals to limit the crowds of people, as well as temporary disclosure of schools and universities, and promoting the use of face masks in public locations. After controlling successfully the first wave of the COVID-19 outbreak with nearly 20 days without reported cases, on 18 March 2020, Vietnam confronted a great challenge when new local cases with unknown causes of transmission were detected in Hanoi, a metropolitan of Vietnam. Until 31 March 2020, 213 new cases were confirmed, and most of them were asymptomatic. During this period, more strict social distancing measures were implemented, including banning crowds of people with more than ten people as well as requesting closure of nonessential places such as educational institutions or entertainment places, restricting the intercity and intracity movement. On 31 March 2020, the Directive 16/CT-TTg was issued which required nationwide social distancing implementation in 15 days. During this period, 59 new cases of COVID-19 were identified (4); however, since 17 April, Vietnam had confirmed no community transmission despite extensive testing. On 23 April, the Vietnam Government decided to loosen the national lockdown and issued the Directive 19/CT-TTg on 24 April about COVID-19 prevention and control strategies in the “new normal” condition, in which social distancing played a major role (5).

**Figure 1:**
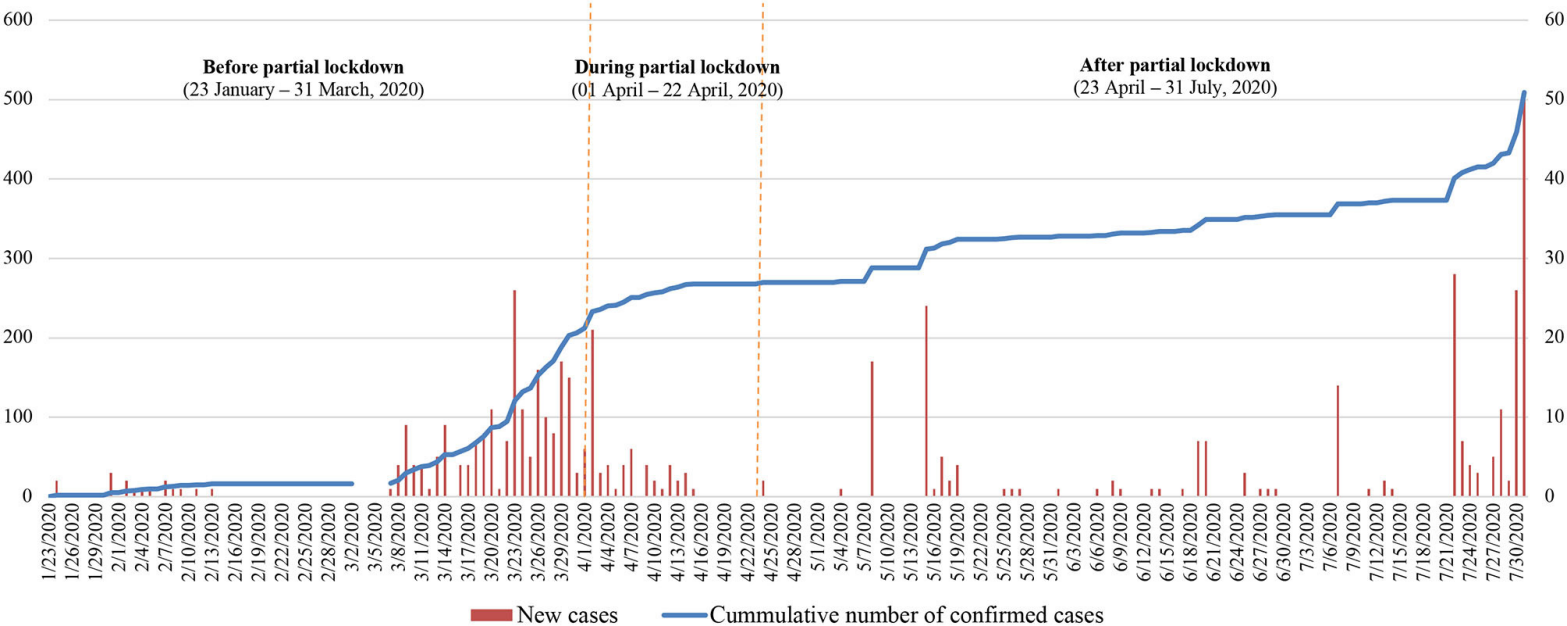
The incidence of confirmed Covid-19 cases and its cumulative numbers, 23 January to 31 July 2020, Vietnam (2).

The high compliance of Vietnamese with social distancing measures can be explained in multiple perspectives. In terms of the leadership, initially, after the detection of the first case, the Vietnam government rapidly formed the Task Force for the COVID-19 epidemic under the direction of the Vice Prime Minister. The Task Force members encompassed leaders of ministries, localities, and press representatives, aiming to develop national COVID-19 preventive and control measures. Previous experiences with Severe Acute Respiratory Syndrome (SARS) and H1N1 epidemics supported the Task Force to quickly decide the key directions for combating COVID-19, in which social distancing measures had a central role (6). Thus, Directive 06/CT-TTg, Directive 16/CT-TTg, and Directive 19/CT-TTg were issued which underlined the social distancing measure implementation to prevent the spread of COVID-19 by maintaining physical distance and reducing social interactions. Moreover, local authorities issued regulations to guide and enforce social distancing in their community. As per these regulations, people not following social distancing recommendations such as going out in unnecessary cases and fleeing from isolation areas or quarantine facilities have to face administrative sanctions. On the other hand, the government maintained and resuscitated economic activity by encouraging Vietnamese businesses to transition to remote work. A continued support system in the provision of essential services and supplies was suggested by the government, aimed to facilitate adherence to social distancing measures for people and communities. People also received daily messages from the Government via mobile phones or mass media to motivate them to adhere to social distancing. In addition, the government protected people from being exposed to fake news about COVID-19 and the effects of social distancing by providing clear and transparent information about COVID-19 in Vietnam (7).

Adherence to social distancing can also be justified by the attributes of Vietnamese people. Recent evidence from a global survey showed that Vietnamese people were highly satisfied with and believed in the government's measures in the prevention and control of COVID-19 (8). This result might be achieved by various activities of the government as discussed above, including the assurance of essential goods during social distancing, clearing of COVID-19 risk communication, and prompting of actions to control the epidemic. Moreover, the prior experience with severe infectious epidemics (e.g., SARS, H1N1) helped people to understand the importance of social distancing in reducing the transmission rate. On top of that, Vietnamese culture might primarily contribute to the success of the social distancing approach in Vietnam. In fact, Confucianism determines the core values of Vietnamese society regarding different aspects such as philosophy, social organization, culture, and economy (9). This influence embraces the individuals' social responsibilities in protecting the health and life of other people in their community over their freedom or liberties (10), which are much different from Western culture. Living with this ideology facilitated the Vietnamese to respond to the COVID-19 epidemic in a solidarity way, that people altogether adhered to the social distancing to mitigate the impacts of COVID-19 (11).

Despite the more and more increasing global Covid-19 patient number with the overload of the health system, Vietnam has still been responding well to the Covid-19 pandemic with the mobilization of the entire political system. We understand that it is very difficult to implement high-level Covid-19 containment measures in the current phase as in the previous phase. In the “new normal” condition after the national lockdown strategy, one of the biggest future challenges we would like to emphasize is the indifference and subjectivity of the people in the prevention and control of COVID-19. Therefore, all ministries, sectors, organizations, and society from central to local levels need to harmoniously combine administrative and specialized solutions to suggest prompt and efficient actions in the worst-case scenarios in Vietnam.

In resource-constrained settings like Vietnam, strategies to promote aggressive social distancing should be based on the effectiveness analysis of this measure in different locations. The elements from the government and from the public contributing to the good compliance with social distance need to be further assessed to reflect each locality's situation. Importantly, in the “new normal” condition after COVID-19, compliance with social distancing in Vietnam will be effective when the measures to closely control and monitor repatriation and immigration via its borders are prioritized.

## Author Contributions

H-LV conceptualized the manuscript. HN reviewed and edited for the final manuscript. All authors synthesized data and related information, wrote the manuscript, and have read and agreed to the published version of the manuscript.

## Conflict of Interest

The authors declare that the research was conducted in the absence of any commercial or financial relationships that could be construed as a potential conflict of interest.
